# Assessing olfactory, memory, social and circadian phenotypes associated with schizophrenia in a genetic model based on Rim

**DOI:** 10.1038/s41398-021-01418-3

**Published:** 2021-05-17

**Authors:** Sergio Hidalgo, Jorge M. Campusano, James J. L. Hodge

**Affiliations:** 1 Departamento de Biología Celular y Molecular, Facultad de Ciencias Biológicas, Pontificia Universidad Católica de Chile, Santiago, Chile; 2 School of Physiology, Pharmacology and Neuroscience, Faculty of Life Science, University of Bristol, Bristol, UK

**Keywords:** Schizophrenia, Molecular neuroscience, Physiology

## Abstract

Schizophrenia shows high heritability and several of the genes associated with this disorder are involved in calcium (Ca^2+^) signalling and synaptic function. One of these is the *Rab-3 interacting molecule-1* (*RIM1*), which has recently been associated with schizophrenia by Genome Wide Association Studies (GWAS). However, its contribution to the pathophysiology of this disorder remains unexplored. In this work, we use *Drosophila* mutants of the orthologue of *RIM1*, *Rim*, to model some aspects of the classical and non-classical symptoms of schizophrenia. *Rim* mutants showed several behavioural features relevant to schizophrenia including social distancing and altered olfactory processing. These defects were accompanied by reduced evoked Ca^2+^ influx and structural changes in the presynaptic terminals sent by the primary olfactory neurons to higher processing centres. In contrast, expression of *Rim-RNAi* in the mushroom bodies (MBs), the main memory centre in flies, spared learning and memory suggesting a differential role of Rim in different synapses. Circadian deficits have been reported in schizophrenia. We observed circadian locomotor activity deficits in *Rim* mutants, revealing a role of Rim in the pacemaker ventral lateral clock neurons (LNvs). These changes were accompanied by impaired day/night remodelling of dorsal terminal synapses from a subpopulation of LNvs and impaired day/night release of the circadian neuropeptide pigment dispersing factor (PDF) from these terminals. Lastly, treatment with the commonly used antipsychotic haloperidol rescued *Rim* locomotor deficits to wildtype. This work characterises the role of Rim in synaptic functions underlying behaviours disrupted in schizophrenia.

## Introduction

Schizophrenia is a common and debilitating mental illness that severely affects the quality of life of patients and their families. Due to the diversity, intensity and complexity of its symptoms, this disorder is poorly understood, diagnosed and treated^1,2^. This psychiatric disease is characterized by the presence of a number of behavioural manifestations classified as positive symptoms, a set of conducts that are considered to be exacerbated in schizophrenia patients (e.g. delusions and hallucinations), and negative symptoms, a group of behaviours that are reduced or absent (e.g. social impairments, anhedonia and blunted affect)^3–5^. Further, impairments in cognition, sleep, circadian rhythms and sensory processing, including olfaction, are also observed in this disorder^6–8^. However, the molecular and physiological mechanisms underlying these deficits remain poorly understood^5^.

The heritability of schizophrenia is one of the highest among complex disorders, ranging from 60% to 80%^9^. Substantial efforts are focused on understanding its genetic basis. Recent GWAS studies have replicated findings from previous studies, identifying genes associated with a higher incidence for schizophrenia, like *CACNA1C*, which encodes the pore-forming α-subunit of the L-type Cav1.2 voltage-gated calcium channel (VGCC)^10,11^. New associations have also been recognized, including the *RIM1* gene, which encodes the presynaptic proteins RIM1α and RIM1β^12^. Although detailed information about the *RIM1* alleles associated with schizophrenia is limited, several reports have linked this gene to other neuropsychiatric disorders, such as autism, in which disruptive mutations (e.g. open-reading frameshifts and insertions) are associated with the disorder^13,14^. RIM1 proteins act as scaffolding molecules in the active zone of presynaptic terminals, clustering VGCCs at the neurotransmitter release sites. They also dock presynaptic vesicles through their interaction with the vesicular protein Rab-3^15,16^. Recent studies have shown impaired sensorimotor gating, increased reactivity to psychotomimetic drugs and reduced social interactions in *RIM1α* homozygotic knock-out mice (*RIM1α*^−/−^), all considered schizophrenia-relevant features^17–19^. Loss of RIM also decreases vesicle tethering at the presynaptic active zone^20^, impairs presynaptic plasticity, with reduced evoked and spontaneous vesicle release frequency^15,21,22^ and generates a compensatory increase in post-synaptic density size^20,23^.

*Drosophila* has served as a model organism to study the contribution of genes to several complex behaviours in humans, and also to diseases, including neurodegenerative and neuropsychiatric disorders, such as schizophrenia^24–28^. So far *Drosophila* studies assessing the function of RIM1 have mostly been conducted at the peripheral and developing larval neuromuscular junction (NMJ). These studies revealed that the *Drosophila* RIM1 ortholog called Rim, showed conserved functions in evoked neurotransmission, VGCC clustering and synaptic plasticity^29,30^. The need for tractable animal models to advance our understanding of schizophrenia pathophysiology makes the *Drosophila Rim* mutant a potentially important tool to gain new insights on the underlying causes of this disorder; however, there is a paucity of knowledge on its behavioural role in adults.

The olfactory and the clock circuits are particularly well characterized and tractable in *Drosophila* and show high molecular, anatomical and functional conservation with their counterparts in mammals^31–34^, thereby facilitating the study of olfactory and circadian phenotypes related to schizophrenia in flies. In the *Drosophila* olfactory circuit, odorants are received by olfactory receptor neurons, which connect with the antennal lobe projection neurons (AL PNs) in the antennal lobe (AL)^35,36^. AL PNs send olfactory information to higher processing centres, namely the MB which generates olfactory memories and the lateral horn (LH) which is important for innate olfactory behaviours^37,38^. Multi-modal sensory information conveyed to both brain regions is also used for the generation of different behavioural outputs^39–42^.

Likewise, the neural circuit that regulates circadian rhythms has been intensively studied in *Drosophila* and is highly tractable, consisting of 75 pairs of clock neurons targetable with defined clock gene promoters^43,44^. This network is composed of dorsal and ventral groups of clock neurons^43,45,46^. The lateral ventral group of neurons, the PDF-containing LNvs, can be divided into the small and large LNvs (s-LNvs and l-LNvs, respectively) with the s-LNvs being the most important in maintaining the behavioural and molecular circadian rhythms under constant darkness (DD)^43,47^. The s-LNvs neurons send projections to the dorsal part of the brain, where they connect with dorsal clock neurons and output circuits of the clock such as the central complex, a fly brain region that controls movement^34,48^. Two important features that seem to contribute to the output of these neurons are the circadian structural plasticity of s-LNvs axons and PDF release from these terminals^34,48^. Firstly, the dorsal axonal arborization of s-LNvs goes from a higher morphological complexity, the open state during the day, with high accumulation of PDF, to a more compact, lower complexity state during the night, with low PDF abundance^43,49^.

In the present study, we manipulated the expression of *Rim* in different neural circuits to assess its contribution to behaviour. Knocking down *Rim* in defined sets of neurons reduced olfactory performance, decreased social behaviour and impaired locomotor circadian rhythmicity. In contrast, lack of *Rim* in memory-associated MB neurons had no effect on memory, suggesting a differential contribution of Rim in different CNS synapses. Structural and functional changes were found to underlie some of the behavioural changes reported and were consistent with Rim functioning in Ca^2+^ signalling and neuropeptide release.

## Materials and methods

### Fly stock

Flies were raised at 25 °C on 12 h light:12 h dark (LD) cycles on a standard corn-yeast meal diet. *CSw*^*−*^ flies (kindly donated by Dr. Scott Waddell, University of Oxford, UK) were crossed with flies bearing *UAS* (*UAS*/*+*) transgenes and used as control genotypes. Due to the high contribution of the genetic background to circadian phenotypes^33^, we have also included the *GAL4* controls (*GAL4*/*+*) for circadian locomotor analysis in Fig. 3. *Tim-GAL4* and *PDF-GAL4*, flies were a gift from Dr. Ralf Stanewsky (University of Münster, Germany). *PDF-GAL4; UAS-Tub:GFP* (*PDF; Tub:GFP*) flies were kindly donated by Dr. Herman Wijnen (University of Southampton, UK). The following strains were obtained from Bloomington *Drosophila* Stock Center (BDSC; stock number provided in brackets): *c309-GAL4* (6906)^50,51^, *OK107-Gal4* (854)^26^*, GH146-GAL4* (30026)^52,53^, *Rim*^*MI03470*^ (37056), *Rim*^*Ex98*^ (78047), *Df(3R)ED5785* (9207, hereon referred to as *Df*) which is a mutant bearing a deficiency in the third chromosome and therefore lacks the *Rim* gene, *UAS-Rim-RNAi* (44541; chromosome II insert, hereon referred to as *Rim-RNAi II*), *UAS-Rim-RNAi* (27300; chromosome III insert, hereon referred to as *Rim-RNAi III*), *UAS-Rim:GFP* (78051), *UAS-GCaMP6f* (42747) and *UAS-eGFP* (5431). The *Rim*^*Ex98*^*/Df* flies were generating by crossing the *Rim*^*Ex98*^ and the *Df* flies, as previously described^30^.

### Behavioural test

#### Olfactory performance using single fly tracking

Experiments were carried out as previously reported^25,54^. Single male flies were placed in a 3.9 cm-diameter circular arena and their behavioural response to an aversive odorant (1% benzaldehyde, Bz, Sigma) was recorded for 3 min. Buritrack software and the Centroid Trajectory Analysis application^55^ were used to generate heat plots of flies behaviour and Fiji software was used to compute an olfactory index (OI) calculated as

$${\rm {OI}} = \frac{{({\rm {AUC}}\,{\rm {H}}_2{\rm {O}} - {\rm {AUC}}\,{\rm {Bz}})}}{{({\rm {AUC}}\,{\rm {H}}_2{\rm {O}} + {\rm {AUC}}\,{\rm {Bz}})}}$$

where AUC means area under the curve in the pixel profile; AUC H_2_O = time spent in the water side; and AUC Bz = time spent in the Bz side. Flies were also recorded for three-min in absence of Bz to quantify centrophobism and locomotion as previously reported^54^, and to discard preference bias within the arena.

### Social space paradigm

Social space experiments were carried out using the same setup described by Simon et al. (2012)^56^. Groups of 30–40 flies were collected 2 days post eclosion and then kept on fresh food at 24 °C overnight. To avoid sexual dimorphism in social space phenotypes and confounding effects of courtship, only male flies were used. After 2 h of acclimatization in the room where the experiment would take place, flies were loaded into the social space arena, consisting in an isosceles triangle with base of 14.5 cm and height of 14.8 cm, giving a free space area of about 86.5 cm^2^. The arena was homemade out of glass and acrylic as previously reported^56,57^. A period of 30 min was given to allow the flies to assume static positions within the arena^56^. Afterwards, a single photo was taken and the distance of each fly to its nearest neighbour was calculated using Fiji software^56,57^.

### Aversive olfactory conditioning

Olfactory memory experiments were performed as previously described^26^. Briefly, groups of 20–50 flies were collected and kept for at least 24 h at 25 °C and 70% relative humidity in the behavioural room to allow acclimatization. On the day of the experiment, flies were transferred into a training tube lined with an electrifiable grid. After 90 s rest, flies were exposed to an odorant (conditioned stimulus, CS^+^) paired with twelve 70 V DC electric shocks (unconditioned stimulus, US) over 60 s. This was followed by a 45 s rest with fresh air. Flies were then exposed to the reciprocal odorant (CS^−^) with no electric shock. Odorants used were either 3-octanol (OCT, Sigma) or 4-methylcyclohexanol (MCH, Sigma). Memory was evaluated 2 min or 1 h post-conditioning to test short-term memory (STM) or intermediate-term memory (ITM), respectively; a performance index (PI) was calculated using the following equation:

$${\rm {PI}} = \frac{{(N_{{\rm {CS}} - } - N_{{\rm {CS}} + })}}{{(N_{{\rm {CS}} - } + N_{{\rm {CS}} + })}}$$

where *N*_CS−_ and *N*_CS+_ is the number of flies choosing CS^−^ and CS^+^, respectively. The CS^+^ odour was reversed in alternate groups of flies to account for innate bias toward one odorant; a *n* = 1 was calculated as the average of the performance of the two groups where CS^+^ odour was reversed. Sensorimotor controls were performed to assess whether electric shock and odorant avoidance were comparable between different strains. Briefly, for olfactory acuity the odorant and air were pumped through opposite arms of the T-maze and flies were allowed to decide between both stimuli for 2 min. For electric shock avoidance, flies are allowed to choose between two training tubes, one of them connected to the stimulator providing electric shocks. The flies that correctly avoided the shock tube over the total number of flies were quantified and reported as percentage avoidance (Table S1).

### *Drosophila* activity monitoring (DAM)

Locomotor activity monitoring experiments were conducted as previously described^58,59^. The activity of 2–5 days old male flies was measured using the DAM system (DAM2, TriKinetics Inc., USA). Flies were transferred into DAM tubes and maintained for 2–5 days under 12:12 LD conditions to assure proper entrainment had taken place, followed by DD, where the first 2 or 5 days were used for circadian rhythm analysis. To assess the rhythm strength, autocorrelation analysis was used following previously published protocols^58,59^. From this analysis, it was possible to obtain a rhythmicity statistic (RS) value, defined as the ratio between the rhythmicity index and the absolute value of the 95% confidence interval in the derived autocorrelogram. By convention, values > 1.5 indicate strong rhythmicity characteristic of a normal wild type fly^59–61^. Additionally, an estimated period length under free-running conditions (i.e. DD without external circadian cues) was calculated from the distance between the autocorrelation peaks (i.e. lag between the peaks)^60^. Flies with RS < 1.5 were excluded from the period calculation^58,59^. All data were analysed in MATLAB using the Sleep and Circadian Analysis MATLAB Program^60^. Only flies that survived until the last day of DD were used for analysis.

### Imaging

#### Calcium imaging

Calcium imaging using the genetically encoded GCaMP6f optogenetic reporter was performed as previously described^26,62^ and adapting protocols for live imaging of [Ca^2+^] fluorescence in presynaptic buttons of AL neurons^63^. Flies were anesthetised under CO_2_, decapitated and their brains dissected in extracellular saline containing (in mM): 101 NaCl, 1 CaCl_2_, 4 MgCl_2_, 3 KCl, 5 d-glucose, 1.25 NaH_2_PO_4_, and 20.7 NaHCO_3_, pH 7.2. Brains were held with the dorsal part facing up, using a custom-made anchor and visualized with a ×40 water-immersion lens on an upright microscope (Zeiss Examiner Z1). Extracellular saline (3 mL/min) was used to perfuse the brains and transient (12 s) bath application of 100 mM potassium chloride (KCl) in extracellular solution was used to transiently depolarize and stimulate the neurons.

Images were acquired at 4 frames/s with 100 ms exposure using a charge-coupled device camera (Zeiss Axiocam) and a 470 nm light-emitting diode light source (Thor Labs). Baseline fluorescence (*F*_0_) was calculated as the mean fluorescence during the first 20 s of recordings (80 images), prior to the start of the high [KCl] perfusion. The change in fluorescence was expressed relative to baseline fluorescence. Data is expressed as [(*F*−*F*_0_)/*F*_0_], where *F* is fluorescence at any given time following high KCl exposure; thus, data is expressed as a metric of transient Ca^2+^ increase. Data were processed and analysed using RStudio version 1.1.463 (RStudio, Inc., Boston, MA).

### Immunohistochemistry and imaging analysis

Immunohistochemistry procedure was conducted as previously described^58^. Brains were dissected at two day-points: 2 h after lights were switched on (zeitgeber (ZT)2, i.e. at 11 a.m.) and 2 h after lights were switched off (ZT14 i.e. 11 p.m.). Samples were fixed in 4% paraformaldehyde (in PBS plus 0.5% Triton X-100) avoiding tissue exposure to white light. Brains were washed and blocked in 5% normal goat serum (NGS, Thermo Fisher Scientific # 50197Z) and then incubated with primary antibodies in 5% NGS at 4 °C for 48 h. To detect PDF a monoclonal antibody specific to the neuropeptide was used (1:200; Developmental Studies Hybridoma Bank, #PDF-C7) and green fluorescent protein (GFP) detection was enhanced by using an anti-GFP antibody (1:1000; Life Technologies #A11122). Vectashield hard set medium (Vector Laboratories) was used as a mounting media for confocal visualization. Confocal stacks images were acquired at 2 μm steps using a Leica TCS SP8 AOBS confocal laser scanning microscope attached to a Leica DMi8 inverted epifluorescence microscope. Axonal arborization of the dorsal projections was quantified using Sholl analysis, with some modifications^49,58,64,65^. Ten evenly spaced (5 μm) concentric rings centred at the first branching of the dorsal projections were drawn and the number of intersections of each projection with the rings was quantified. To assess PDF intensity within the clusters a homemade Fiji macro was used defining the fluorescence intensity in each PDF cluster. Scoring of both, axonal arborization and PDF intensity was automatized and blind to avoid observer bias. Data recorded in the two hemispheres in a brain was averaged and that number was reported as the result for that animal (*n* = 1).

### Drug exposure for behavioural assay

Drug exposure was conducted as previously described^66^. Haloperidol (H1512, Merck) was dissolved in 100% ethanol to generate a stock solution and a 1 mM concentration in food was achieved by mixing the required volume in molten fly food. Flies were given the haloperidol as adults four days prior and throughout the behavioural experiment. Circadian and locomotor activity data for these experiments was obtained by using the ShinyR-DAM app^67^. When carrying out these experiments we noticed a difference in fly survival after the 3rd day in DD conditions in mutant and control flies fed haloperidol compared to control conditions (mutant and control flies fed vehicle; not shown). Therefore, in this particular experiment, we only report data collected during 2 consecutive days in DD conditions. Morning anticipation was calculated as the difference between the averaged activity in ZT21.5–24 and ZT17–19.5 and evening anticipation was calculated as the difference between the averaged activity in ZT9.5–12 and ZT5–7.5^58^.

### Statistical analysis

No sample size calculation was carried out. No randomization was performed to carry out experiments. Analysis for data was not performed blindly. No data exclusion criteria were pre-determined. All statistical analyses were performed using GraphPad Prism (version 8.00, GraphPad Software, La Jolla, CA, USA). Datasets were scrutinized for normal distribution using the Shapiro–Wilk test in order to choose the appropriate parametric or non-parametric analysis to be used. Data are presented as mean ± standard error of the mean (SEM), of the *n* (number of animals, trials, brains) indicated in each figure legend. Data presented was acquired from animals/brains obtained from different crosses/vials, and therefore are biological replicates. Statistical tests used for each comparison are detailed in figure legends. Also, statistical descriptions for each comparison are provided throughout the manuscript. Statistical levels are denoted as following *p < 0.05, **p < 0.01 and ***p < 0.001.

## Results

### Effect of Rim knockdown in olfactory processing and social behaviour

Olfactory impairments are widely reported in patients with schizophrenia and are known to be a prodromal symptom of frontal and temporal-limbic disorders related to schizophrenia^8,68,69^. We used *Drosophila* to assess olfactory acuity by tracking behaviour of single flies exposed to the aversive odorant benzaldehyde, Bz. Initially, we used a previously described *Rim* mutant, *Rim*^*Ex98*^, which lacks part of the *Rim* gene including the PDZ and C2 domains, over a deficiency chromosome (*Df*) lacking the entire *Rim* gene^30^.

**Fig. 1:**
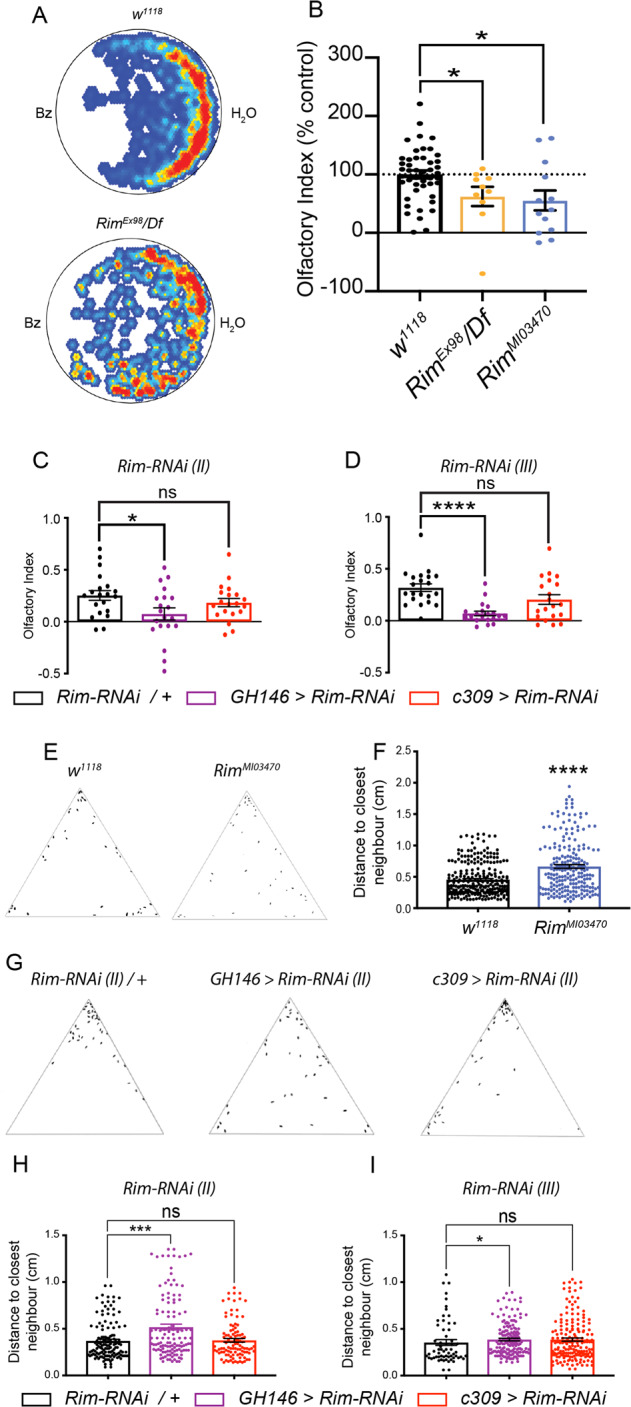
Loss of *Rim* function in the olfactory system caused impaired social interaction. **A** Averaged heat-maps showing the position of control (upper plot) and *Rim*^*Ex98*^*/Df* mutant (lower plot) flies exposed to benzaldehyde (Bz). **B** A reduction in the olfactory index was observed in the mutants (orange open bar) compared to control flies (black open bar; one-way ANOVA with Holm–Sidak post-hoc test, *p* = 0.0333). Similar results are observed in an independent loss of function mutant, *Rim*^*MI03470*^ (open blue bar) compared to control flies (*p* = 0.0116). **C** Targeted knockdown of *Rim* in the antennal lobe (*GH146-Gal4*; purple bars) but not mushroom body (*c309-Gal4*; red bars) neurons resulted in reduced olfactory index as compared to the control (black open bars). **D** Similar results were observed using an independent *RNAi* transgene to *Rim* (one-way ANOVA with Dunnett’s *post-hoc* test). Data is presented as mean ± standard error of the mean (SEM) in this and all subsequent histograms, with each dot representing an independent measurement (henceforth in all figures), *n* (*w*^*1118*^) = 46, *n* (*Rim*^*Ex98*^*/Df*) = 10, *n* (*Rim*^*MI03470*^*)* = 13, *n* (*Rim-RNAi (II)*/*+*) = 20, *n* (*Rim-RNAi (III)*/*+*) = 21, *n* (*GH146>Rim-RNAi (II)*) = 20, *n* (*GH146>Rim-RNAi (III*)) = 20, *n* (*c309>Rim-RNAi (II)*) = 20 and *n* (*c309 > Rim-RNAi (III))* = 20 flies). **E** Male flies were placed in a triangular arena and the distance between each was measured to determine their social space. Representative results for control (left) and *Rim*^*MI03470*^ flies (right) are shown. **F** An increase in social distance was observed in *Rim* mutant flies. **G** Representative results are shown for control flies (left) and for antennal lobe (*GH146-Gal4*, middle) or mushroom body (*c309*-*Gal4*, right) neuron-specific knockdown of *Rim*. These manipulations revealed only a change in social space in *GH146>Rim-RNAi* flies. **H** and **I** An increase in social space, defined by the distance of a fly to its closest neighbour, is observed when either of two different *Rim-RNAi* was expressed the antennal lobe (purple bars in **H** and **I**) as opposed to the situation when the *RNAi* is expressed in the mushroom body (red bars), compared to control (black bars). Data analysed with an unpaired *t*-test (**F**) and by a Kruskal–Wallis test followed by Dunn’s multiple comparisons test (**H** and **I**). *n* = 3–5 repetitions in each condition with 34–40 flies for each.

*Rim*^*Ex98*^*/Df* mutants showed less reactivity to the odorant compared to control flies (Fig. 1A, bottom and top panels, respectively). Quantification of the effect showed a reduction in the olfactory index (OI) in this mutant (*F*(2, 66) = 5.412, *p* = 0.0067, Fig. 1B). We also characterized flies that carried a transposon insertion in the 23rd exon of the *Rim* gene (*Mi{MIC}* insertion*; Rim*^*MI03470*^). This second loss of function allele of *Rim* also displayed a reduction in the OI (Fig. 1B, open blue bar). In addition, flies with targeted reduction of *Rim* in the AL PNs (*GH146>Rim-RNAi (II)*) also displayed a reduction in the OI to about 70% that is seen in controls (*F*(2, 57) = 3.399, *p* < 0.05, Fig. 1C). Results were further confirmed using an independent *RNAi* line against a non-overlapping region of *Rim* (*GH146>Rim-RNAi (III)*, *F*(2, 58) = 11.75, *p* < 0.0001, Fig. 1D). In contrast, knocking down *Rim* in the MBs did not influence the olfactory performance of flies with either of the two *RNAi* lines tested (*c309>Rim-RNAi (II)*, *p* = 0.50; *c309>Rim-RNAi (III)*, *p* > 0.05, Fig. 1C, D).

As the olfactory system has been shown to be a key mediator of social behaviour in different animals^70–72^, we tested whether flies that displayed impaired olfaction are also deficient in social space behaviour. To do this, we used a social space paradigm^56^ in which flies are kept in a closed arena for 30 min before assessing the distance of each fly to its closest neighbour.

While control animals formed groups within the arena, *Rim*^*MI03470*^ flies failed to do so (Fig. 1E), as reflected by an increase in distance between each fly and its nearest neighbour (Mann–Whitney *U* test, hereafter *U* = 21418, *p* < 0.0001, Fig. 1F). Knocking down *Rim* in the AL PNs (Fig. 1G) resulted in a similar defect in clustering behaviour as *Rim*^*MI03470*^. A 1.4-fold increase in social distance was observed in Rim deficient flies compared to control (Kruskal–Wallis statistic *H*, hereafter *H* = 14.06, *p* < 0.0001, Fig. 1H). A similar effect was observed using a different *RNAi* strain (*H* = 7.254, *p* < 0.05, Fig. 1I). Knocking down *Rim* in the MBs with either *RNAi* did not result in a change in distance to the closest neighbour compared to control flies. No differences were found in single-fly locomotion or centrophobism, after knocking-down *Rim* in the AL or MB. This suggests that the differences observed in these experiments arise from social impairments as opposed to locomotor or centrophobism deficits (Supplemental Fig. S1).

Although *Rim* knockdown in the MBs does not affect either Bz aversion or social space, we went onto investigate the role of Rim in olfactory learning and memory, which is known to be mediated by this structure^39,73,74^. Using the aversive olfactory conditioning paradigm, memory performance was tested at 2 min and 1 h after training to test for STM and ITM, respectively^26,62^. No effect was observed on STM (*F*(4, 27) = 1.382, *p* = 0.27, Supplemental Fig. S2) or ITM (*F*(4, 23) = 1.492, *p* = 0.24, Supplemental Fig. S2) when knocking down Rim in the MB (*c309>Rim-RNAi*). Similar results were found when using another GAL4 driver that expresses throughout the MB (*OK107-Gal4*^26^) (STM: *F*(4, 18) = 2.725, *p* = 0.06, ITM: *H* = 2.035, *p* = 0.36, Supplemental Fig. S2). Therefore, Rim expression in the MBs is not required for any of these behaviours.

### Rim loss-of-function affects the structure and function of presynaptic terminals of AL PNs innervating the LH

**Fig. 2:**
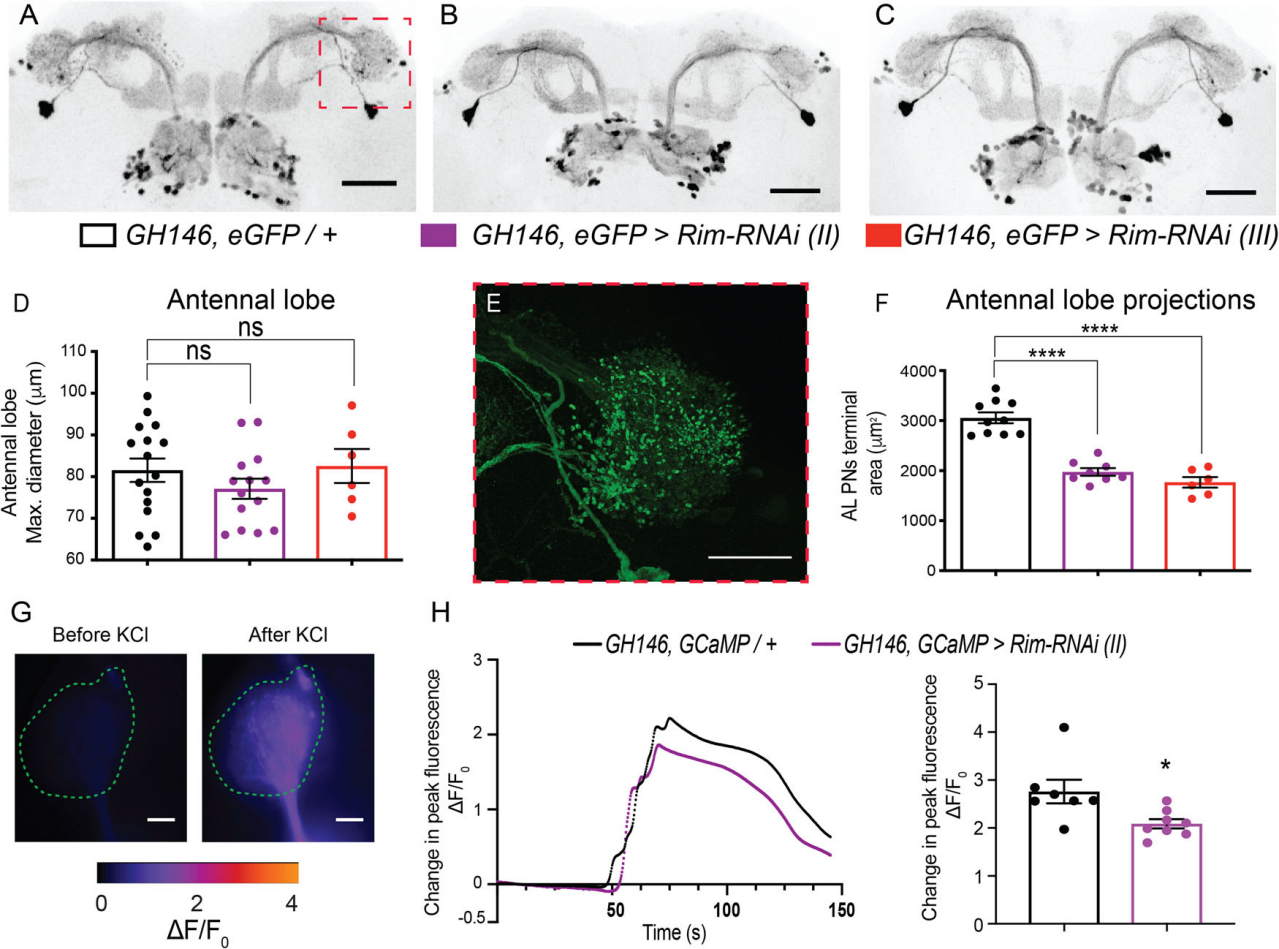
*Rim* knockdown altered the structure and function of antennal lobe projection neuron (AL PN) terminals on to the lateral horn (LH). Representative images of GFP expression in the antennal lobe projection neurons (AL PNs) of **A** control (*GH146, eGFP*/*+*); **B** *GH146, eGFP>Rim-RNAi (II)*; and **C** *GH146, eGFP>Rim-RNAi (III)* showed no apparent gross morphological difference. Scale bars represent 50 μm. **D** Knocking-down *Rim* in the projection neurons did not affect the antennal lobe structure as maximum diameter remained unchanged when compared to control flies. **E** A representative confocal image of the terminals reaching the lateral horn from AL PNs labelled by *GH146, eGFP* (from red square in **A**). Scale bar represents 20 μm. **F** AL PNs *Rim* knockdown caused a reduction in the area covered by these terminals to about half that of controls. Data were analysed with one-way ANOVA followed by a Dunnett’s post hoc test. *n* (*GH146, eGFP*/*+*) = 16, *n* (*GH146, eGFP>Rim-RNAi (II)*) = 14 and *n* (*GH146, eGFP>Rim-RNAi (III)*) = 6 brains. **G** Basal fluorescence in AL PN terminals on to LH was low under basal conditions (left panel) while exposure to a depolarizing, high concentration of potassium chloride (KCl) causes an increase in fluorescence (right panel). Calibration bar showing the changes in fluorescence can be observed below. Scale bars show 10 μm. **H** high [KCl]-induced calcium transients were observed in control flies and a reduced response was detected in *Rim* knockdown flies; both responses eventually returned to baseline after high [KCl] was washed out (mean fluorescence as solid lines). A reduction in the amplitude of the peak fluorescence was found upon *Rim* knockdown (Mann–Whitney test). *n* (*GH146; GCaMP*/*+*) = 7 and *n* (*GH146; GCaMP>Rim-RNAi (II)*) = 8 brains.

Given the effects of *Rim* knockdown in AL PNs on olfaction and social behaviour, we looked for any underlying structural and/or functional defects of these neurons. However, no gross-morphological alterations were found when the inner antenno-cerebral tract or the median antenno-cerebral tract pathways were studied in knockdown and control animals (Fig. 2A–C, Supplemental Fig. S3). Furthermore, similar AL diameter (*F*(2, 33) = 0.9314, *p* = 0.40, Fig. 2D) and an equal number of labelled cells were observed (*U* = 21, *p* = 0.72, Supplemental Fig. S3) between strains. Nonetheless, close inspection of the presynaptic terminals reaching the LH (Fig. 2E) showed an approximate 50% reduction of the area covered by the AL PNs terminals in *Rim* knockdowns compared to control (*F*(2, 21) = 50.03, *p* < 0.0001, Fig. 2F).

In order to determine if these changes in synaptic morphology were accompanied by changes in neural activity, the genetically encoded calcium sensor, GCaMP6f, was expressed in the AL PNs and high [KCl] evoked Ca^2+^ transients were assessed in terminals of *Rim* knockdown versus control (Fig. 2G). Bath application of depolarizing high KCl evoked a robust signal in normalized fluorescence, that decayed once it was washed out in both control and knockdown neurons (Fig. 2H, traces in left panel). Quantification of independent experiments revealed a reduction in peak amplitude in *Rim* knockdown compared to control flies (*U* = 6.5, *p* < 0.05, Fig. 2H, right panel).

### Rim contribution to circadian locomotor activity under constant darkness

In order to determine the role of Rim in circadian rhythms, locomotor activity was measured in constant darkness (referred as to DD). As opposed to control flies whose behaviour appeared rhythmic under constant-dark conditions (Supplemental Fig. S4), *Rim*^*Ex98*^*/Df* flies appeared less rhythmic in DD (Supplemental Fig. S4).

**Fig. 3:**
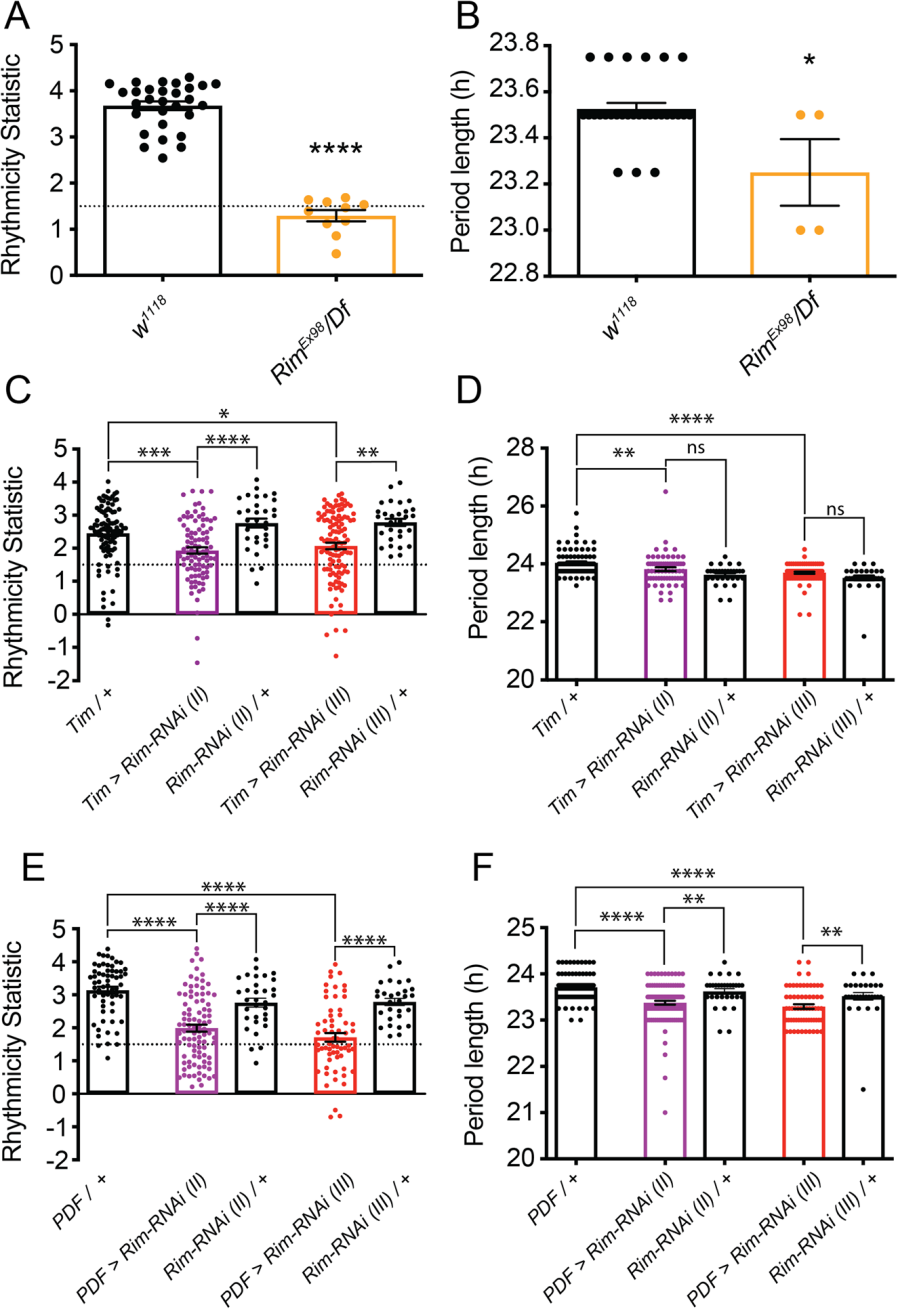
*Rim* knockdown in the clock network reduced rhythmicity and shortened period length under constant darkness (DD). The rhythmicity statistic (RS), as a measure of rhythm strength and the free-running period length of the circadian rhythm, was determined over 5 days in DD. **A** *Rim*^*Ex98*^*/Df* mutants showed a reduction in RS (orange bar; the dotted line shows RS = 1.5, flies with RS above that line are considered rhythmic), and **B** shorter period (orange bar) compared to control flies (open black bars). **C** To map this phenotype, *Rim* was knocked down throughout the clock, resulting in a significant reduction in rhythm strength and **D** shortening of the period compared to *Tim*/*+* control. **E** Restricted expression of *Rim-RNAi* to just a dozen PDF-positive LNvs replicated the reduction in rhythm strength and **F** lengthening of the period in *PDF>Rim-RNAi* compared to control flies. Unpaired *t*-test was conducted for data in **A** and **B**. Kruskal–Wallis test was conducted for data in **C**, **D** and **F** with Dunn’s post hoc test. One-way ANOVA with Holm–Sidak’s post hoc test. *n* (*w*^*1118*^) = 29, *n* (*Rim*^*Ex98*^*/Df*) = 10, *n* (*Tim*/*+*) = 95, *n* (*Tim>Rim-RNAi (II)*) = 92, *n* (*Tim>Rim-RNAi (III)*) = 114, *n* (*PDF*/*+*) = 61, *n* (*PDF>Rim-RNAi (II)*) = 94, *n* (*PDF>Rim-RNAi (III)*) = 63, *n* (*Rim-RNAi (II)*/*+*) = 32 and *n* (*Rim-RNAi (II)*/*+*) = 29 flies.

Autocorrelation analysis revealed a reduction in the RS, a measure of the strength of the behaviour rhythms of about 3-times in *Rim*^*Ex98*^*/Df* mutants compared to control flies (Fig. 3A). 100% of control flies showed the expected wildtype rhythmic behaviour, this was in contrast with *Rim*^*Ex98*^*/Df* mutants where only 58% of flies were rhythmic (Table S2). A reduction in the period length was observed in a portion of *Rim*^*Ex98*^*/Df* rhythmic flies compared to controls (Fig. 3B). Similar reductions in rhythmic behaviour resulted from *Rim* knockdown throughout the clock circuit using *timeless (tim)-Gal4*^44,75^ (*Tim>Rim-RNAi*, Supplemental Fig. S4), resulting in a reduction in rhythm strength to ~50% of that of controls (Fig. 3C, Table S2). There appeared to be a reduction in the period length in *Tim>Rim-RNAi* compared to *Tim*/*+* control, but not when compared to the *UAS*/*+* controls (Fig. 3D). Moreover, we observed an unexpected difference between the *Gal4*/*+* and *UAS*/*+* control lines. In order to clarify the potential inconsistency between genotypes as well as to map the *Rim* circadian phenotypes to subpopulations of neurons in the clock circuit*, Rim-RNAi* expression was restricted to just the LNvs using the clock-neuron-specific *PDF-Gal4*^44,76^ (*PDF>Rim-RNAi*, Supplemental Fig. S4). Consistent with the *Rim*^*Ex98*^*/Df* and *Tim>Rim-RNAi* arrhythmic behaviour, knocking down *Rim* only in the LNvs reduced rhythm strength (Fig. 3E), with only 66% and 51% of the *PDF>Rim-RNAi* flies being rhythmic compared to a 100% and 90% of controls (Table S2). Moreover, a comparison of the period of the rhythmic flies in each group again revealed a reduction in period length in *PDF>Rim-RNAi* flies (Fig. 3F).

### Rim knockdown affects circadian-dependent remodelling of pacemaker s-LNvs terminals and PDF cycling

**Fig. 4:**
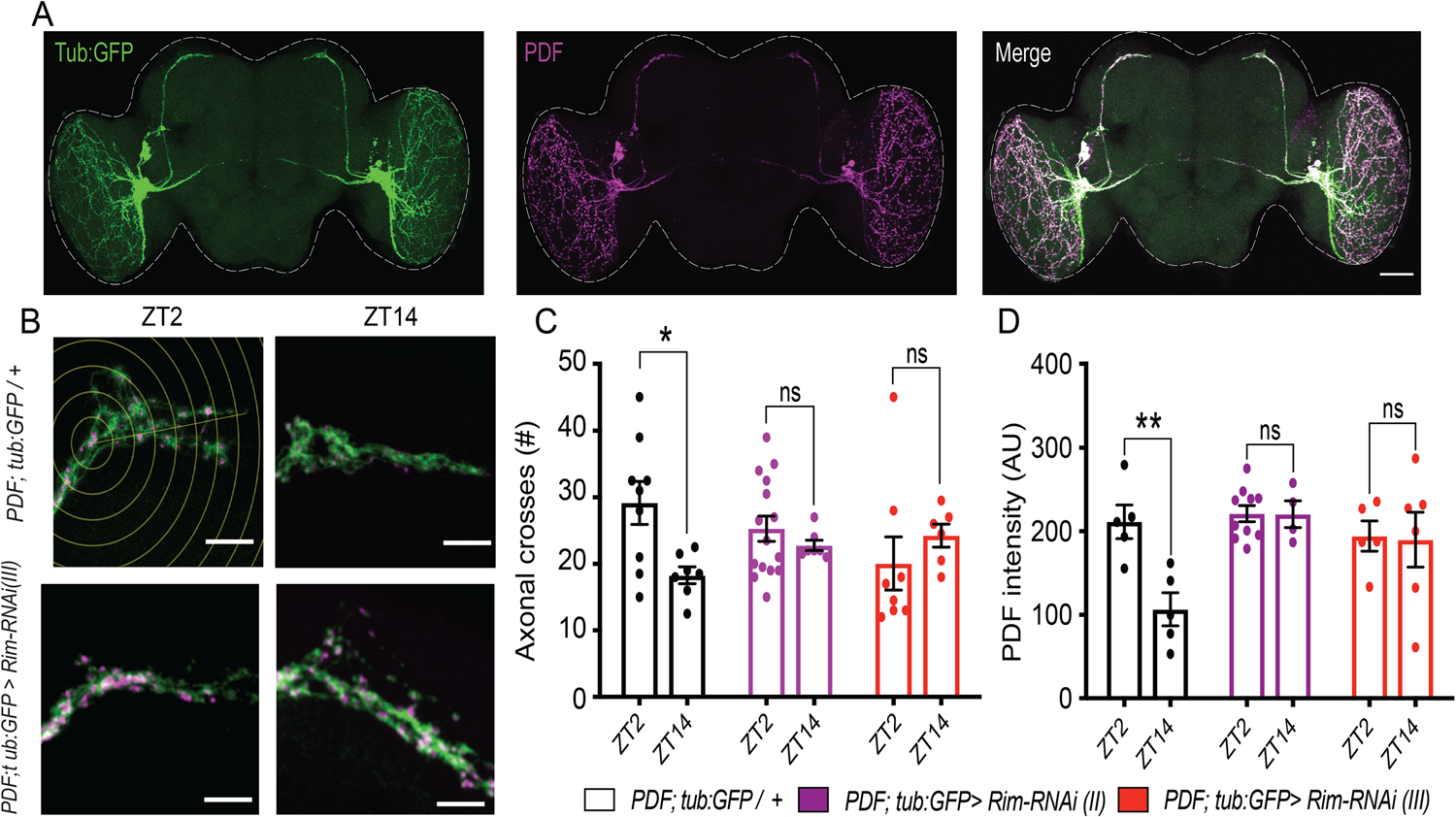
Day/night remodelling of dorsal s-LNvs terminals and PDF availability were removed by *Rim* knockdown. **A** *PDF-GAL4* driven expression of a GFP-tagged version of tubulin (Tub:GFP) was used to label all the neuronal processes of LNvs. Tub:GFP (green) can be observed in the terminals, axons and somas of LNvs (left panel). Immunodetection of PDF is shown in magenta (middle panel). Merged images show colocalization of l-LNvs terminals and PDF immunoreactive signals (right panel). Scale bar represents 50 μm. **B** Representative images of s-LNvs dorsal terminals during daytime (ZT2, i.e. 11 a.m.) and night (ZT14, i.e. 11 p.m.) in control flies (top panels) and *PDF; tub:GFP>Rim-RNAi* (bottom panels). Scale bars show 10 μm. White lines in the upper left panel in **B** represent the circles used for Sholl analysis. **C** Analysis revealed that there were significantly more axonal crosses for control flies at day than at night (open black bars). This day/night difference in terminal complexity was not observed in the *PDF>Rim-RNAi* flies (purple and red bars show results using two independent RNAi lines). **D** PDF was also found to be cycling in control flies (open black bars), with high levels during the day (ZT2) and reduced levels at night (ZT14) as measured by the mean intensity of each PDF cluster. This day/night difference in PDF accumulation was not detected in *PDF>Rim-RNAi* flies (purple and red bars), which remained in a perpetually high state. Data in **C** and **D** were analysed using two-way ANOVA with Sidak’s post hoc (*PDF; tub:GFP*/*+*) = 7–9 brains, *n* (*PDF; tub:GFP>Rim-RNAi (II)*) = 7–15 brains, *n* (*PDF; tub:GFP>Rim-RNAi (III)*) = 8–6 brains.

Given the changes in rhythmic behaviour in *Rim* mutants, we studied whether *Rim* knockdown had an effect on the structural plasticity of s-LNvs. A GFP-tagged version of tubulin expressed in the LNvs allow the visualization of l-LNvs projections to the medulla and the s-LNvs dorsal and ventral projections, which co-localized with the PDF peptide assessed by immunohistochemistry (Fig. 4A). Dorsal terminals from s-LNvs in control flies displayed day/night remodelling, as previously described^58,64^, with a complex axonal arborization during the day which was significantly more branched compared to at night (Fig. 4B, top left and right panels, respectively). However, *Rim* knockdown in the LNvs removed this day/night difference in terminal complexity (Fig. 4B, bottom left and right panels), resulting in an intermediate level of complexity between control day and night levels (Fig. 4C).

In order to assess whether Rim was involved in the rhythmic accumulation of PDF in s-LNvs terminals, PDF levels were quantified at these two-time points. PDF signal intensity was measured in each PDF marked area within the dorsal terminals of the s-LNvs, making the quantification of structural remodelling and changes in PDF accumulation independent of one another (Supplemental Fig. S5). PDF shows circadian cycling in control flies, as previously described^75^, with high levels of PDF at ZT2 and lower levels at ZT14. In contrast, PDF accumulated to similar levels at ZT2 and ZT14 in *PDF>Rim-RNAi* (Fig. 4D).

**Fig. 5:**
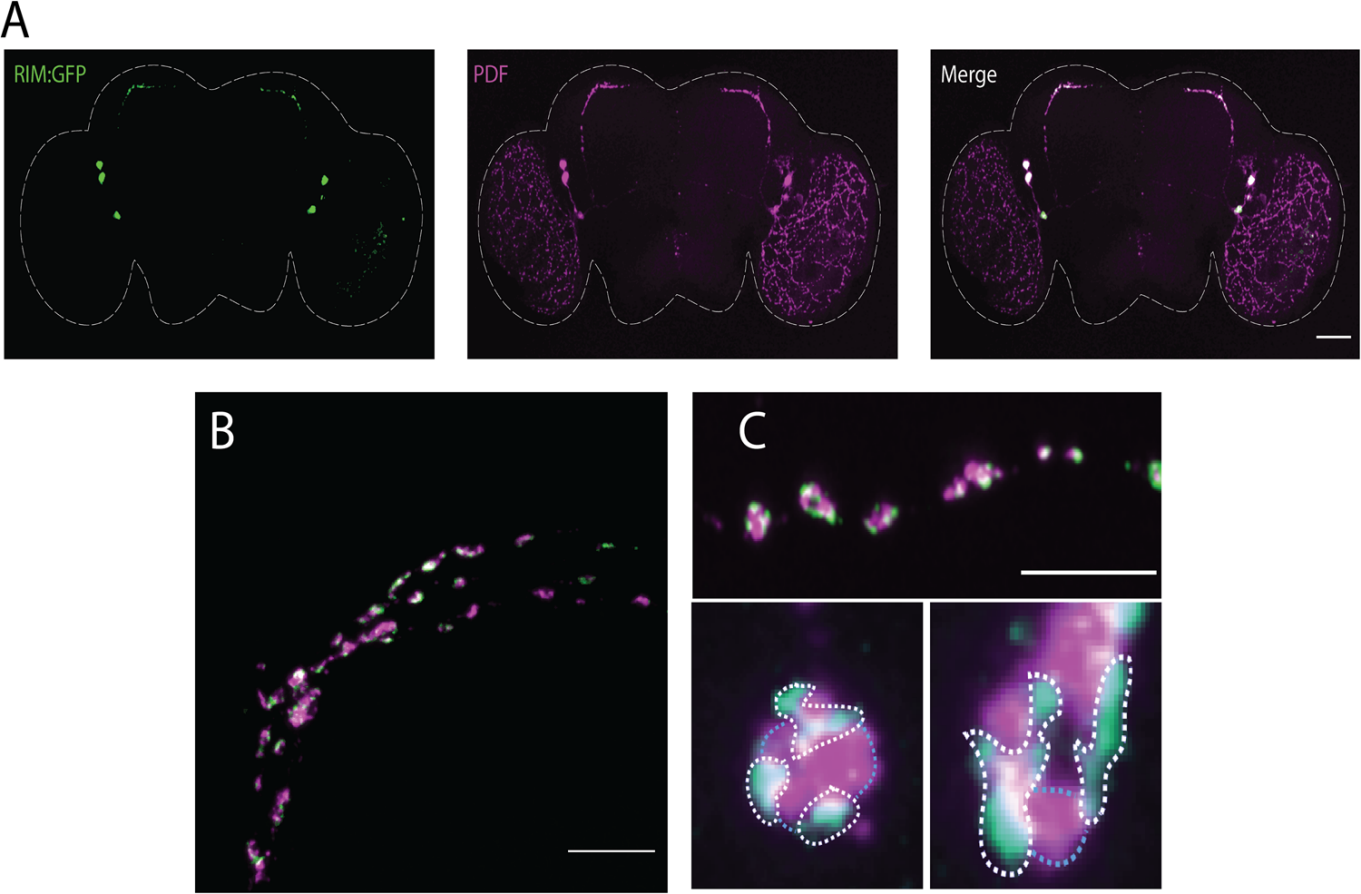
Rim:GFP was found near but not with PDF in s-LNvs dorsal terminals. **A** Expression of a GFP-tagged version of Rim was driven in the LNvs using the *PDF-GAL4* driver (in the green, left panel) while PDF was assessed by immunohistochemistry (in magenta, central panel). A merge of images is observed at the right panel. Distribution of Rim:GFP was found to be mostly localized to the soma of the small and large LNvs while also detected in the dorsal projection of the s-LNvs. **B** Higher magnification of the sLNv terminals showed Rim:GFP and PDF were not completely colocalized. **C** Rim:GFP (green) was occasionally found to co-localize (white) with PDF (magenta). Closer inspection of high magnification images (bottom panels) revealed that Rim:GFP formed aggregates (identified by white dotted line), mostly not co-localizing with PDF (shown in light blue dotted line). Scale bar is 50 μm in **A** and 10 μm in **B** and **C**.

In order to determine if Rim co-localized with PDF in the s-LNvs terminals, a GFP-tagged version of Rim was expressed using a *PDF* driver and PDF localization was determined by immunohistochemistry. This revealed that Rim:GFP was preferentially localized in the s-LNvs terminals as well as in the soma, whereas it appeared to be absent in the l-LNvs terminals terminating in the medulla (Fig. 5A). Interestingly, co-localization images showed PDF was in close proximity to Rim:GFP but they were not directly co-localized (Fig. 5B). Higher magnification images of the PDF containing terminals revealed that Rim:GFP formed aggregates around the sites where PDF might be released, lending further support to Rim regulating PDF release (Fig. 5C).

### Pharmacological effect of the antipsychotic haloperidol on Rim mutant phenotypes

**Fig. 6:**
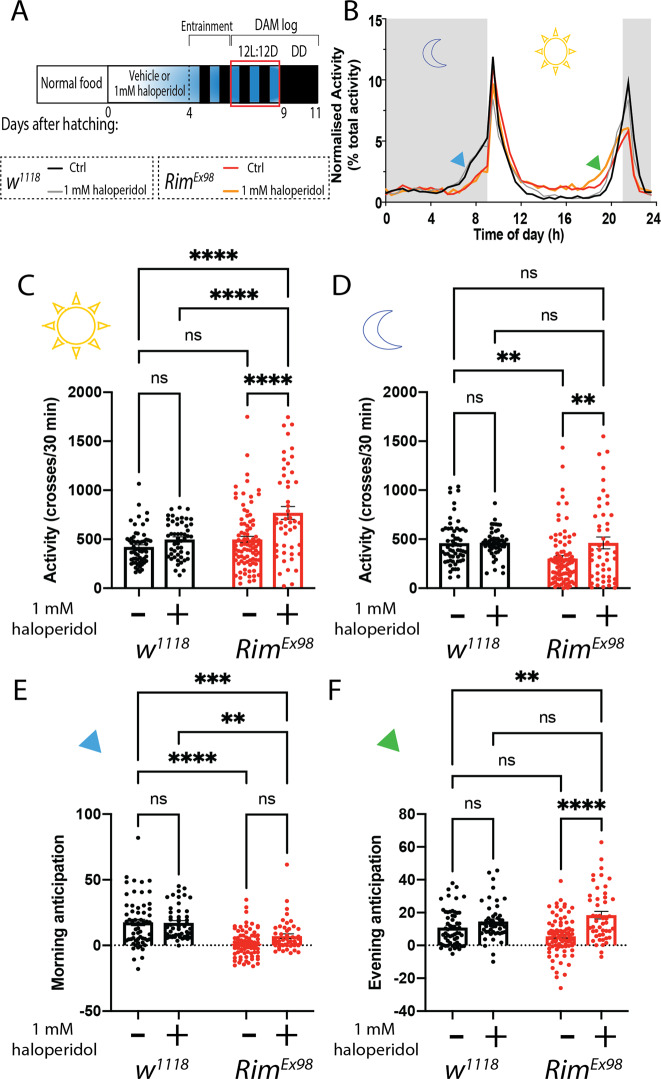
Haloperidol treatment rescues night-time activity under light–dark conditions in *Rim* mutants. **A** Newly eclosed flies were fed with the antipsychotic drug haloperidol (1 mM) for 4 days prior to and then continuously throughout the behavioural test. Animals were entrained for 2 full days in 12:12 LD cycles, followed by 3 days on LD where locomotor circadian activity was measured (red box). **B** Averaged normalized activity profiles of *w*^*1118*^ fed with normal or 1 mM haloperidol-supplemented food (black and grey line, respectively) and *Rim*^*Ex98*^ fed with normal or 1 mM haloperidol-supplemented food (red and orange, respectively) are shown. Grey background shading represents dark period (i.e., night-time) while white background segment represents the light phase (i.e., day-time). Morning and evening anticipation are indicated with blue and green arrowheads, respectively. **C** Day-time activity was not different in *Rim*^*Ex98*^ as compared to control flies; however, haloperidol treatment increased day-time activity in mutants, an effect that is not observed in control flies. **D** Night-time activity is reduced in *Rim*^*Ex98*^ *as* compared to control flies. The deficit observed in mutant animals is reversed by 1 mM haloperidol treatment with no effect observed in control flies. **E** Morning anticipation, calculated as the difference between the averaged activity in ZT21.5–24 and ZT17–19.5, is decreased in *Rim*^*Ex98*^ flies regardless if flies are treated or not with haloperidol. **F** Evening anticipation, calculated as the difference between the averaged activity in ZT9.5–12 and ZT5–7.5^58^, is not different in *Rim*^*Ex98*^ mutants compared to control flies. Feeding flies with haloperidol increases the evening anticipation in mutants while no changes are observed in control flies under the same conditions. Data were analysed using two-way ANOVA followed by Sidak’s multiple comparison test. *n* (*w*^*1118*^ Ctrl) = 59 flies, *n* (*w*^*1118*^ 1 mM haloperidol) = 50 flies, *n* (*Rim*^*Ex98*^ Ctrl) = 88 flies and *n* (*Rim*^*Ex98*^ 1 mM haloperidol) = 48 flies.

In order to provide pharmacological validation of our *Drosophila Rim* model, we analysed the effect of a typical antipsychotic, haloperidol^66,77^, on circadian locomotor activity. Flies were collected after hatching and then exposed for 4 days to 1 mM haloperidol in regular food. Afterward, animals were entrained for 2 full days in 12L:12D cycles. Then, locomotor activity was monitored for 3 days in LD conditions, followed by 5 days in DD. Flies were maintained on food supplemented with drug for the entire procedure, while in the control condition animals were maintained in regular food (Fig. 6A).

As it has been reported a disruption in day and night locomotor activity in schizophrenia^78^, we sought to determine if similar deficits occur in our model and whether these alterations could be affected by haloperidol treatment. Therefore, we measured the average daily locomotor activity of flies in LD (Fig. 6B). Under these conditions, daytime activity in *Rim*^*Ex98*^ homozygotes was not different from that recorded in control flies. While haloperidol treatment did not have an effect on daytime activity in control flies, it induced an increase in this parameter in *Rim*^*Ex98*^ animals (*F* (1, 252) = 11.12, *p* = 0.001, Fig. 6C). On the other hand, night-time activity was reduced in the *Rim*^*Ex98*^ flies when compared to control animals. Treatment with haloperidol restored the night activity to control levels in the mutants. However, it did not show any effect on *w*^*1118*^ control flies.

From the activity profiles showed in Fig. 6B, a reduction in the morning anticipation can be observed in *Rim*^*Ex98*^ flies when compared to control animals (Fig. 6B, red and black traces). This effect can be quantified by comparing the average morning anticipation of control and mutant flies (*F*(1, 254) = 68.67, *p* < 0.0001, Fig. 6E). Haloperidol treatment did not affect the average morning anticipation in either genotype (*F*(1, 254) = 0.1560, *p* = 0.69). On the other hand, the evening anticipation was not different between *w*^*1118*^ and *Rim*^*Ex98*^ flies. Interestingly, there was an increase in evening anticipation in mutants treated with haloperidol, an effect not observed in control flies (*F*(1, 254) = 68.67, *p* < 0.0001, Fig. 6F).

**Fig. 7:**
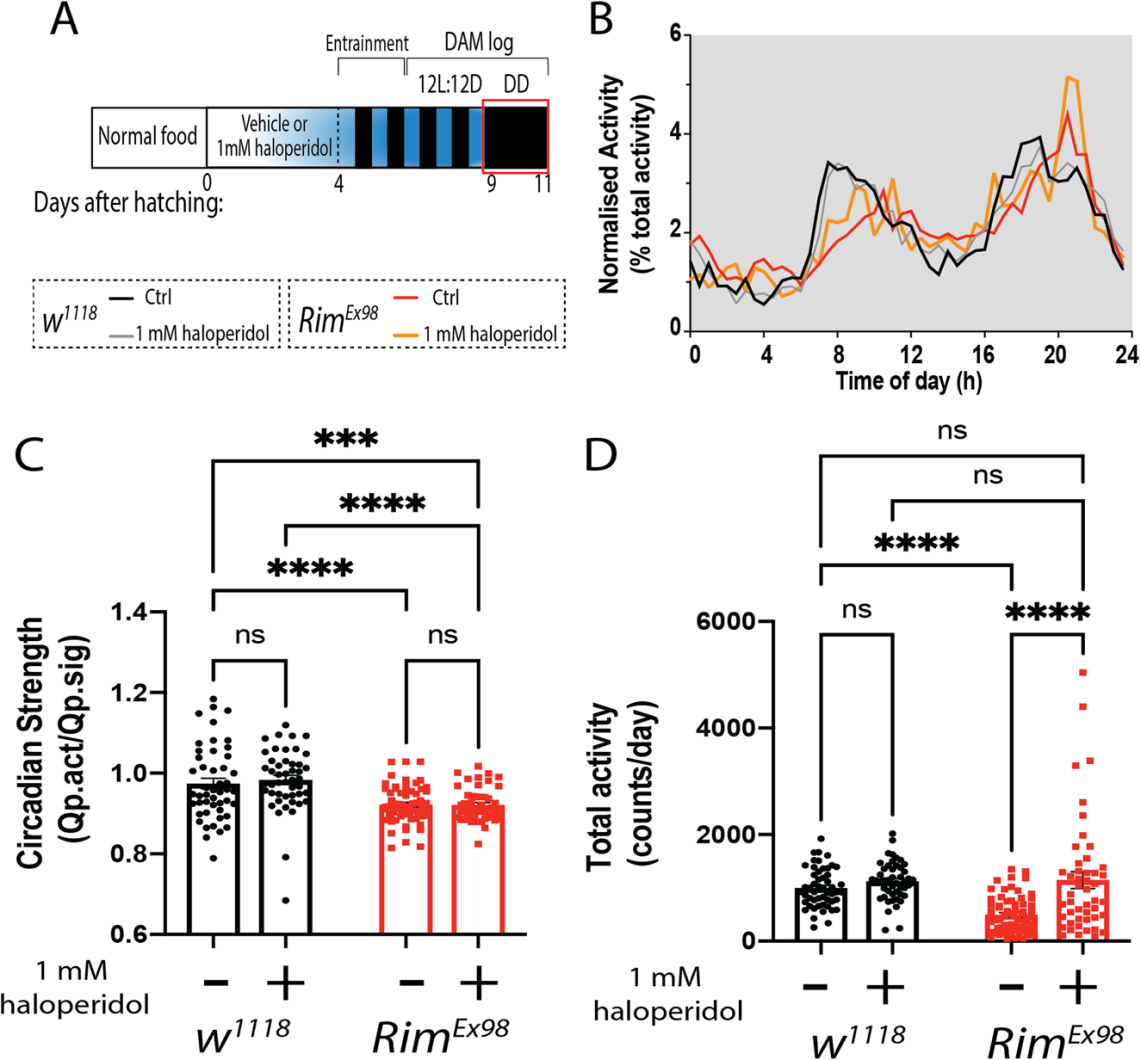
Haloperidol treatment rescues total locomotor activity but not rhythmicity recorded in constant darkness in *Rim* mutants. **A** Newly eclosed flies fed with haloperidol (1 mM) were entrained for 2 days in 12:12 LD cycles, followed by 3 days in LD and 2 days in DD, where locomotor circadian activity was measured (red box). **B** Normalized averaged activity profiles of *w*^*1118*^ flies fed with normal (black line) and 1 mM haloperidol-supplemented food (grey line), and *Rim*^*Ex98*^ flies fed with normal (red line) and haloperidol-supplemented food (orange line), in DD conditions. **C** Circadian strength was reduced in *Rim*^*Ex98*^ flies compared to control flies fed with normal food. Haloperidol treatment did not change the circadian strength of *w*^*1118*^ or *Rim*^*Ex98*^. **D** The average locomotor activity between the 2 days in DD was quantified as counts (beam breaks) per day for each fly. *Rim*^*Ex98*^ mutants displayed a reduction in total locomotion compared to *w*^*1118*^ flies in normal food. This decrement was absent in *Rim*^*Ex98*^ flies fed with haloperidol, being rescued to levels indistinguishable from control flies. Data were analysed using two-way ANOVA with Sidak’s post hoc test *n* (*w*^*1118*^ Ctrl) = 59 flies, *n* (*w*^*1118*^ 1 mM haloperidol) = 50 flies, *n* (*Rim*^*Ex98*^ Ctrl) = 88 flies and *n* (*Rim*^*Ex98*^ 1 mM haloperidol) = 48 flies.

We also analysed the effect of haloperidol treatment on the circadian defects we described in *Rim* mutants in DD conditions (Fig. 7A, red box). As shown for the mutant and knockdown animals (Fig. 3), *Rim*^*Ex98*^ homozygotes displayed reduced rhythmicity strength under DD compared to control flies (*F*(1, 207) = 38.94, *p* < 0.0001, Fig. 7B, C). Haloperidol treatment did not affect circadian strength (*F*(1, 207) = 0.1713, *p* = 0.679) in control (*p* = 0.976) or mutant flies (*p* = 0.976) and did not rescue the circadian defect observed in *Rim*^*Ex98*^ flies (Fig. 7C).

Finally, we observed a reduction in locomotor activity in *Rim*^*Ex98*^ flies as compared to control flies under DD conditions (*F*(1, 211) = 8.387, *p* = 0.004, Fig. 7E). Noteworthy, haloperidol treatment (*F*(1, 211) = 22.03, *p* < 0.0001) restored *Rim*^*Ex98*^ locomotion to levels indistinguishable from control flies (*w*^*1118*^_ctrl_ 997.91 ± 51.29 vs. *Rim*^*Ex98*^_haloperidol_ 1146.23 ± 155.83 counts/day, *p* > 0.05). The chemical did not affect locomotor activity in control flies (*w*^*1118*^_ctrl_ 997.91 ± 51.29 vs. *w*^*1118*^_haloperidol_ 1121.14 ± 53.90 counts/day, *p* = 0.736).

## Discussion

Schizophrenia is commonly diagnosed by the presence of different symptoms, including psychosis and asociality. Little is known about the underlying molecular mechanisms^5,79^. Drugs currently used only treat some of the symptoms observed in a percentage of patients. Thus, animal models based on the genetic changes observed in schizophrenia continue to be a powerful tool to uncover the pathophysiology of the disorder and to study new potential targets for therapeutic intervention^80–82^. Here we demonstrated that the *Drosophila* orthologue of the schizophrenia-associated gene *RIM1*, *Rim*, has an important role in distinct neuronal populations mediating different behaviours relevant to the disease.

*Rim* loss of function mutants and animals where *Rim* expression was knocked down in the AL PNs, showed impaired olfactory acuity to the normally highly aversive odorant benzaldehyde. Reduced olfaction is commonly observed in schizophrenia patients^83^. Several studies have established that the threshold for odour detection is decreased in patients by a mechanism that is independent of environmental factors such as the use of neuroleptics or smoking^84–86^. Although well documented, it is not fully understood how olfactory impairments arise in schizophrenia. However, since they occur at the early stages of the disorder, they have been proposed to be a prodromal marker of the disease^83^. Hence, the *Drosophila* olfactory system offers a simple platform to evaluate olfactory dysfunction resulting from mutations in schizophrenia-associated genes.

Previous work has shown that expression in mice of the schizophrenia-associated human splice isoforms of the G72/G30 overlapping genes resulted in impaired smell^87^. Moreover, a null mutation in the schizophrenia-associated gene ortholog *dtnbp-1 (dysb*^1^*)*, showed impaired olfactory habituation in flies, an effect that was mimicked by knocking down *dysb* expression in the AL PNs^88^. It is not known whether mutations in *RIM1* can cause similar olfactory impairments in humans; however, its high expression in the olfactory bulb is consistent with the idea that it could play a role in olfaction^89^.

Impaired social behaviour is a hallmark of schizophrenia, with animal models of this disorder showing phenotypes resembling such deficits in social interaction^4,82^. There are many levels at which social impairments can manifest in people with schizophrenia, including reduced social skills, shyness and disrupted social communication, which can often lead to social withdrawal^4,82^. Studies have reported that one specific aspect of social interaction, namely social space, is a good descriptor of social withdrawal in schizophrenia^90^. Social space is understood as a space where a person interacts with others, and strikingly, schizophrenia patients consistently exhibited increased social space regardless of the different setups used to study this symptom^90–93^. Here we found that social behaviour was affected in *Rim* mutants resulting in them also showing an increase in social space. Similar social impairments were described in *RIM1α*^−/−^ mutant mouse^17,22^. In particular, *RIM1α*^−/−^ mutant mice display decreased time engaged in social recognition of novel mice^17^ and also exhibit deficits in maternal behaviour^22^.

Interestingly, a relationship between olfactory performance and social behaviour has been proposed for schizophrenia and other neuropsychiatric disorders like autism in humans^94–96^. Our results show that the fly social space deficits are also mediated by the olfactory system as they occurred when *Rim* was just knocked down in the olfactory AL PNs. Recent evidence in ants suggested that nesting, a social grouping behaviour, also relies on olfactory processing^97^, in agreement with the idea that similar mechanisms regulate social behaviour in flies. It is known that olfactory information is sent by the AL PNs to two major areas: the MB and the LH^98^. While LH mediates innate olfactory behaviours, the MB participates in odour learning^99,100^. Interestingly, *Rim* knockdown in the AL PNs resulted in prominent structural and functional changes in the terminals reaching the LH. These terminals were smaller and displayed reduced Ca^2+^ responses to depolarizing high [KCl] stimulation.

The LH is known to have a highly defined neuronal structure^101^; therefore it would be interesting to determine if there are any *Rim*-mediated reductions in the AL projections to particular regions of the LH as opposed to a general loss of neuronal connectivity. Based on our images, we were not able to test if this was the case. Our data did suggest that social space information is being processed through the olfactory system and is mediated by the LH with no effect seen with *Rim* knockdown in MBs. We also did not see a decrease in olfactory learning and memory in these flies, even though memory and cognitive impairments have been described in schizophrenia^6,102^. Studies in mammals have also been equivocal in describing the potential role of RIM in memory. While *RIM1α*^−/−^ mice showed impaired memory in fear-conditioning test^18^, deletion of *RIM1* in the dentate gyrus or pyramidal neurons of the CA3 regions resulted in normal memory. This is a puzzling observation since the hippocampus is known to be critical for this type of memory^19^. Therefore, the lack of effect described here is consistent with mammalian studies showing that RIMs can have distinct roles in different types of synapses^103^. Rim expression has not currently been characterized in the fly brain, and to our knowledge, there remains no functioning antibody for such studies; therefore the reason why there are no phenotypes due to knockdown of *Rim* in the MB could be that the gene is not expressed there.

Sleep and circadian disruption are also common manifestations of schizophrenia, affecting up to 80% of individuals with this disorder^104,105^. However, the symptoms are highly heterogeneous, with changes in total sleep, sleep activity patterns and circadian misalignment being the most frequently observed^7,104,105^. Consistently, reduced rhythmicity and shorter free-running period length were observed in *Rim* mutants, phenotypes replicated when *Rim* knockdown was restricted to the LNvs. These neurons have been described as the major pacemakers under DD, sustaining locomotor rhythmicity^43,106^ with flies lacking these PDF-containing neurons exhibiting shorter periods and a lack of rhythmicity^107^. Likewise, *pdf*-null flies also display arrhythmicity under the same conditions^108^. Hence, our results agree with the notion that Rim and the LNvs are important in maintaining rhythmicity under DD^34,43,59,106^.

Another consistent feature of schizophrenia pathology is changes in synaptic and neuronal structure^5,109–111^, with patients showing reduced cortical thickness^109,112^ and decreased brain volume^110,111^. Intriguingly, we also found changes in synaptic and neuronal structure related to the behavioural deficits of the Rim mutants. As shown for the AL PNs, *Rim* expression in the LNvs was necessary to support the synaptic structure of the s-LNvs dorsal terminals. Normally the dorsal projections of the s-LNvs undergo circadian structural plasticity in their axonal branching which goes from a higher terminal complexity during the day, to reduced complexity during the night^49,58,64^. This structural reorganization is accompanied by changes in the number of active zones^64^ and PDF release^34,49,75^, allowing the s-LNvs to potentially change how they interact with postsynaptic clock neurons during different times of the day^64^. Because of this, it is suggested that these clock outputs could be fundamental for the accurate expression of circadian activity. This is supported by our results, as the behavioural phenotypes in the *PDF>Rim-RNAi* flies, were accompanied by loss of this remodelling and accumulation of PDF at perpetually high levels throughout the day and night. Although there is a lack of information on the molecular machinery required for the release of PDF^113^, these data are consistent with Rim regulating PDF release from the s-LNvs dorsal terminals. A recently published study showed that RIM1/2 and RAB3 are essential for neuropeptide release from dense core vesicles^114^, further supporting the idea that Rim might regulate PDF release from the s-LNvs. Moreover, we show that PDF and Rim were localized to dorsal s-LNvs synaptic terminals, with Rim surrounding clusters of PDF neuropeptide. Remarkably, a similar organization of peptidergic vesicles is observed in different neuronal populations, where dense-core vesicles are in close proximity to the presynaptic releasing site^113^, further supporting this idea. Additionally, it is possible that the loss of circadian structural plasticity observed upon *Rim* knockdown might arise from the same impairment of PDF release. Herrero et al. recently showed that PDF is required for this process, through local action of the peptide^115^. Interestingly, *Rim*^*Ex98*^ displayed reduced morning anticipation (Fig. 7) possibly arising from a reduced PDF expression or terminal accumulation that has been shown to be essential for this component of the locomotor circadian activity^45,116^.

Antipsychotic drugs have been used in the treatment of schizophrenia and mood disorders for several years^117^. The antagonist of the dopamine receptor D2 was the first type to be described and is considered now typical antipsychotics^81^. Although they are effective in treating the positive symptoms of schizophrenia, they have several adverse effects that are associated with extrapyramidal action of the drugs^117–119^. Hence, there is a need to developing new therapeutic tools to address this problem and easy ways to test them. Here we show that feeding adult flies with haloperidol was sufficient to rescue *Rim* mutant locomotion deficits to normal levels under constant darkness. This result validates our model for high throughput in vivo testing of novel compounds that reverse this behavioural deficit, which similarly occurs in people with schizophrenia^78^. Although the treatment was sufficient to increase the locomotion in mutant flies, it did not have an effect on circadian strength. It is possible that the drug did not have any impact on this behaviour because, to the best of our knowledge, D2 receptors are not involved in fly circadian rhythms. Also, it has been shown that other dopamine receptors underlie phenotypes observed in *Drosophila* schizophrenia models^120^. It is known, however, that dopamine does have a role in clock output^48^. Therefore, future studies would be useful to clarify if different durations and doses of haloperidol might rescue all the phenotypes including circadian, olfactory or social activity in *Rim* and other *Drosophila* models of the disorder.

In summary, we have shown that loss of function of the fly ortholog of the schizophrenia-associated gene *RIM1* in different neuronal populations of the central brain leads to a range of behavioural deficits. Similar to mice models and consistent with human studies, this *Drosophila* model displayed impaired social behaviour and olfactory performance, likely due to the requirement of Rim for normal structure and Ca^2+^ responses of AL PN presynaptic terminals onto the LH. Likewise, circadian deficits have been associated with schizophrenia. We also found *Rim* mutants exhibited circadian defects including a loss of circadian rhythmicity and decreased period length phenotypes that mapped to the s-LNvs pacemaker neurons. Moreover, loss of *Rim* expression resulted in a loss of day/night differences in s-LNvs dorsal terminal complexity and PDF synaptic abundance. Finally, we showed daily locomotor activity of *Rim*^*Ex98*^ flies was reduced in constant darkness a phenotype that could be reversed by the common schizophrenia treatment, haloperidol, providing pharmacological validation of our model.

Further studies should be conducted to explore whether similar phenotypes are observed with other schizophrenia-associated genes in *Drosophila*. We have recently described that the *Drosophila dysb*^*1*^ mutant displays similar olfactory and social deficits as the ones here described for *Rim*^57^, further supporting the use of *Drosophila* mutants to test schizophrenia pathophysiology. In addition, new drug treatments could be tested in this model, for instance, to compare their efficiency with currently available treatments.

## Supplementary information

Supplemental material
