# Supplementary material for: Assessing olfactory, memory, social and circadian phenotypes associated with schizophrenia in a genetic model based on Rim
Source: Transl Psychiatry. 2021 May 17;11:292. doi: 10.1038/s41398-021-01418-3 (PMC8128896; doi:10.1038/s41398-021-01418-3)
Supplement: Supplementary file 1 — Supplemental material [file 41398_2021_1418_MOESM1_ESM.docx]

**Supplemental material.**

**Table S1. Olfactory and shock reactivity controls for the memory assay.** There were no significant differences in avoidance of shock, 3-octanol (Oct) or 4-methylcyclohexanol (MCH) between genotypes.

| genotype | % Shock avoidance  (mean ± SEM) | Oct avoidance (mean ± SEM) | MCH avoidance (mean ± SEM) |
| --- | --- | --- | --- |
| *OK107 / +* | 72.4 ± 4.0 | 0.46 ± 0.07 | 0.67 ± 0.03 |
| *c309 / +* | 85.5 ± 5.4 | 0.40 ± 0.17 | 0.84 ± 0.08 |
| *Rim-RNAi (II) / +* | 91.5 ± 4.1 | 0.46 ± 0.20 | 0.54 ± 0.16 |
| *Rim-RNAi (III) / +* | 80.0 ± 4.3 | 0.48 ± 0.15 | 0.34 ± 0.11 |
| *c309 > Rim-RNAi (II)* | 84.1 ± 3.7 | 0.33 ± 0.14 | 0.43 ± 0.14 |
| *c309 > Rim-RNAi (III)* | 74.3 ± 6.3 | 0.57 ± 0.11 | 0.52 ± 0.10 |
| *OK107 > Rim-RNAi (II)* | 88.5 ± 1.2 | 0.44 ± 0.07 | 0.47 ± 0.12 |
| *OK107 > Rim-RNAi (III)* | 79.9 ± 7.0 | 0.64 ± 0.13 | 0.62 ± 0.04 |


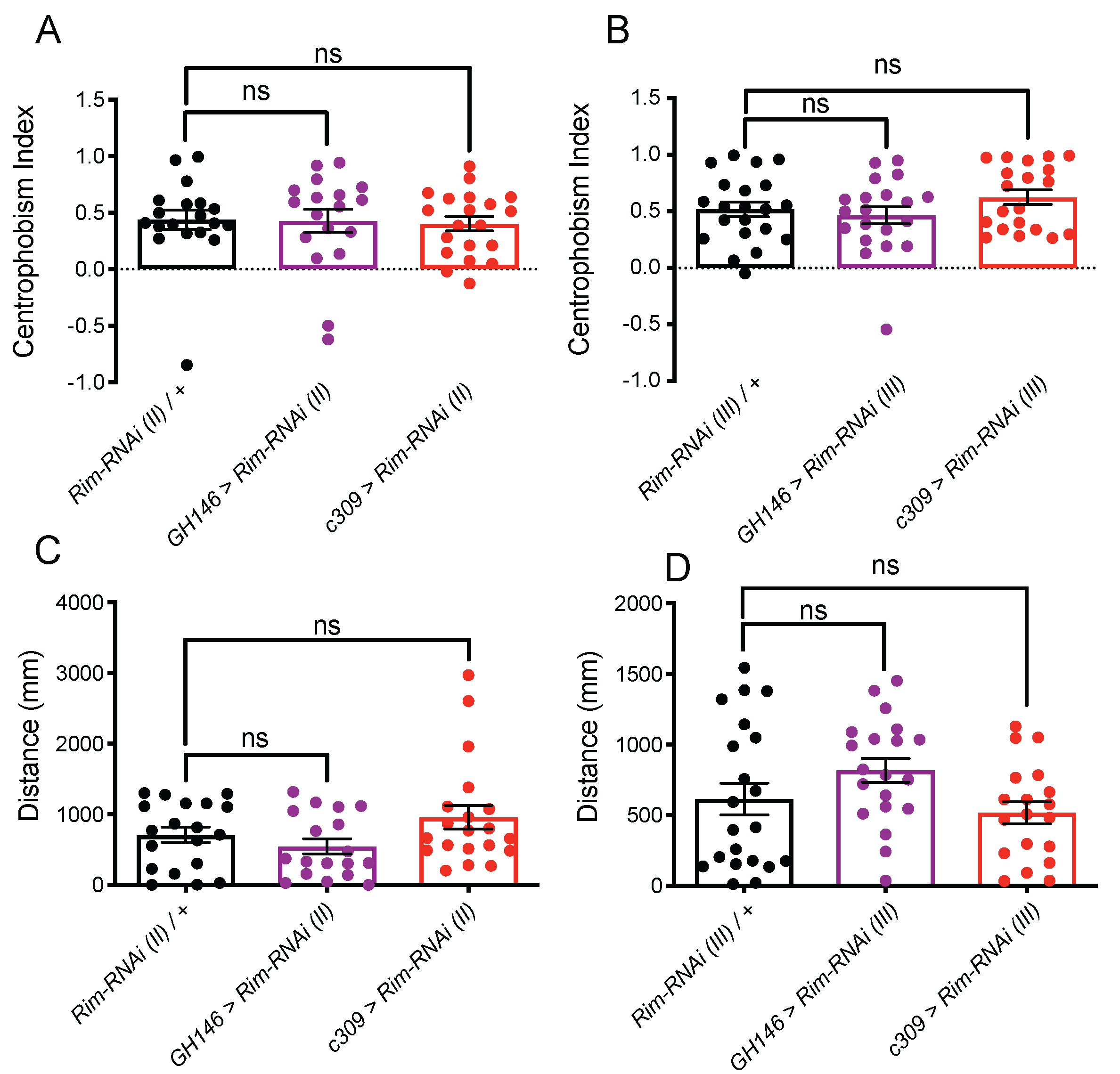


**Fig. S1.** Reduced *Rim* expression in antennal lobe or mushroom body does not alter centrophobism or motor performance**.** The fly’s behaviour was recorded for a 3-min period. Centrophobism index and the total distance travelled were assessed. A, Centrophobism was unchanged in *GH146 > Rim-RNAi (II)* (open purple bars) and in *c309 > Rim-RNAi (II)* (open red bars), compared to control flies (open black bars). B, Similar findings were replicated using a second independent *RNAi* line, *Rim-RNAi (III)*. C, Total distance travelled during the 3 min recording also appeared unchanged in *GH146 > Rim-RNAi (II)* (open purple bars) and in *c309 > Rim-RNAi (II)* (open red bars), compared to control flies (open grey bars). D, Similar results were obtained using *Rim-RNAi (III)* line (D). Data were analysed using one-way ANOVA with Dunn's *post hoc* test. n (*Rim-RNAi (II) / +*) = 20, n (*Rim-RNAi (III) / +*) = 21, n (*GH146 > Rim-RNAi (II)*) = 20, n (*GH146 > Rim-RNAi (III)*) = 20, n (*c309 > Rim-RNAi (II)*) = 20, n (*c309 > Rim-RNAi (III)) =* 20 flies.


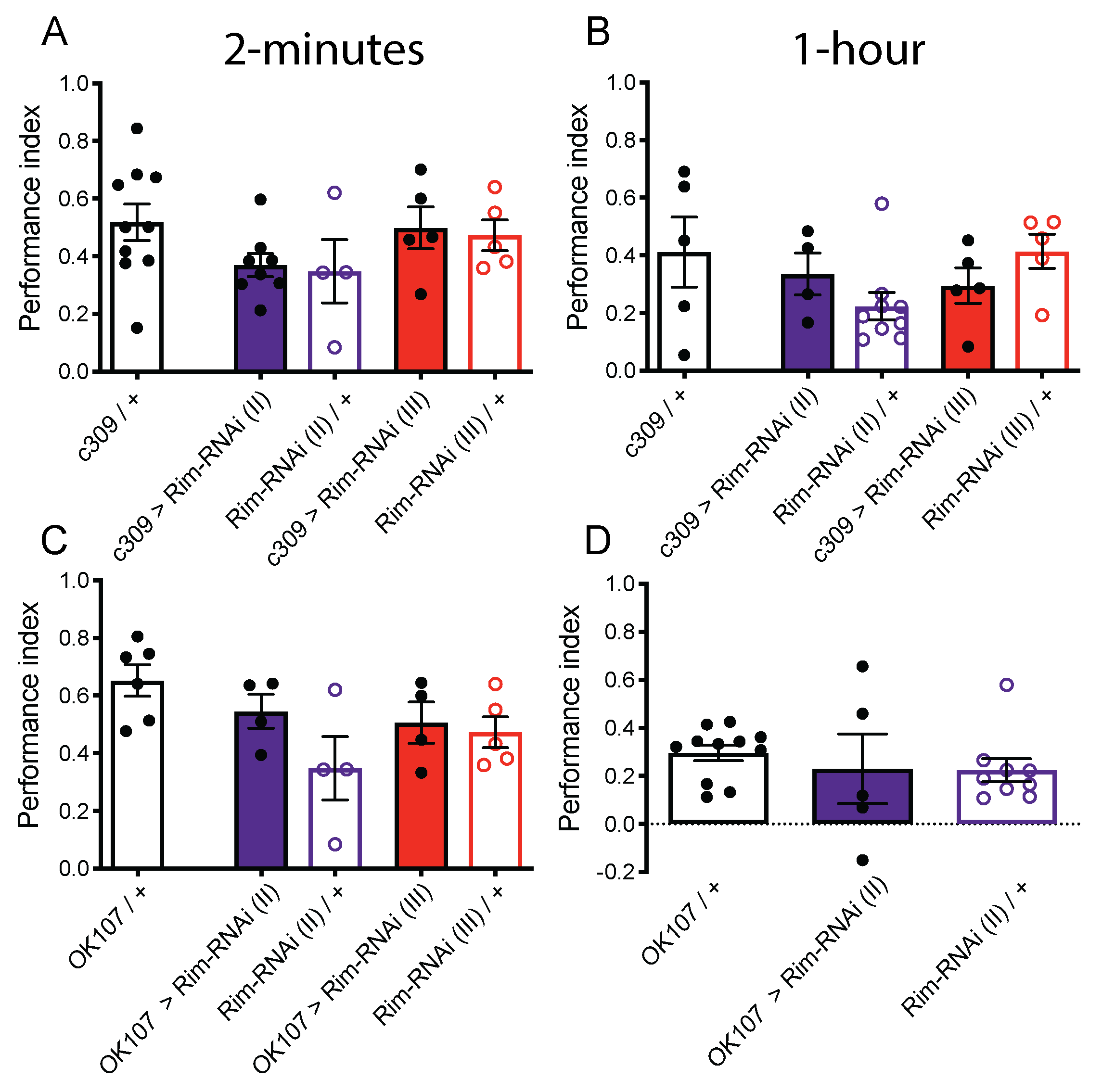


**Fig. S2,** *Rim* knockdown in the mushroom bodies does not affect aversive olfactory learning and memory. Olfactory shock conditioning was used to test memory in flies with expressing *Rim-RNAi*  using the *c309-GAL4* MB driver this had no effect on A, 2 min memory (short-term memory, STM), or B, 1 h memory (intermediate-term memory, ITM) ccompared to control (One-way ANOVA with Sidak’s *post hoc* test). Similar results were replicated with an independent MB driver *OK107-GAL4* with no change in STM (C) and ITM (D) Kruskal-Wallis test with Dunn’s *post hoc* test. n > 4 repetitions (each n consisted of ~100 flies) for each genotype and condition. All comparison where not significant, p > 0.05.

**
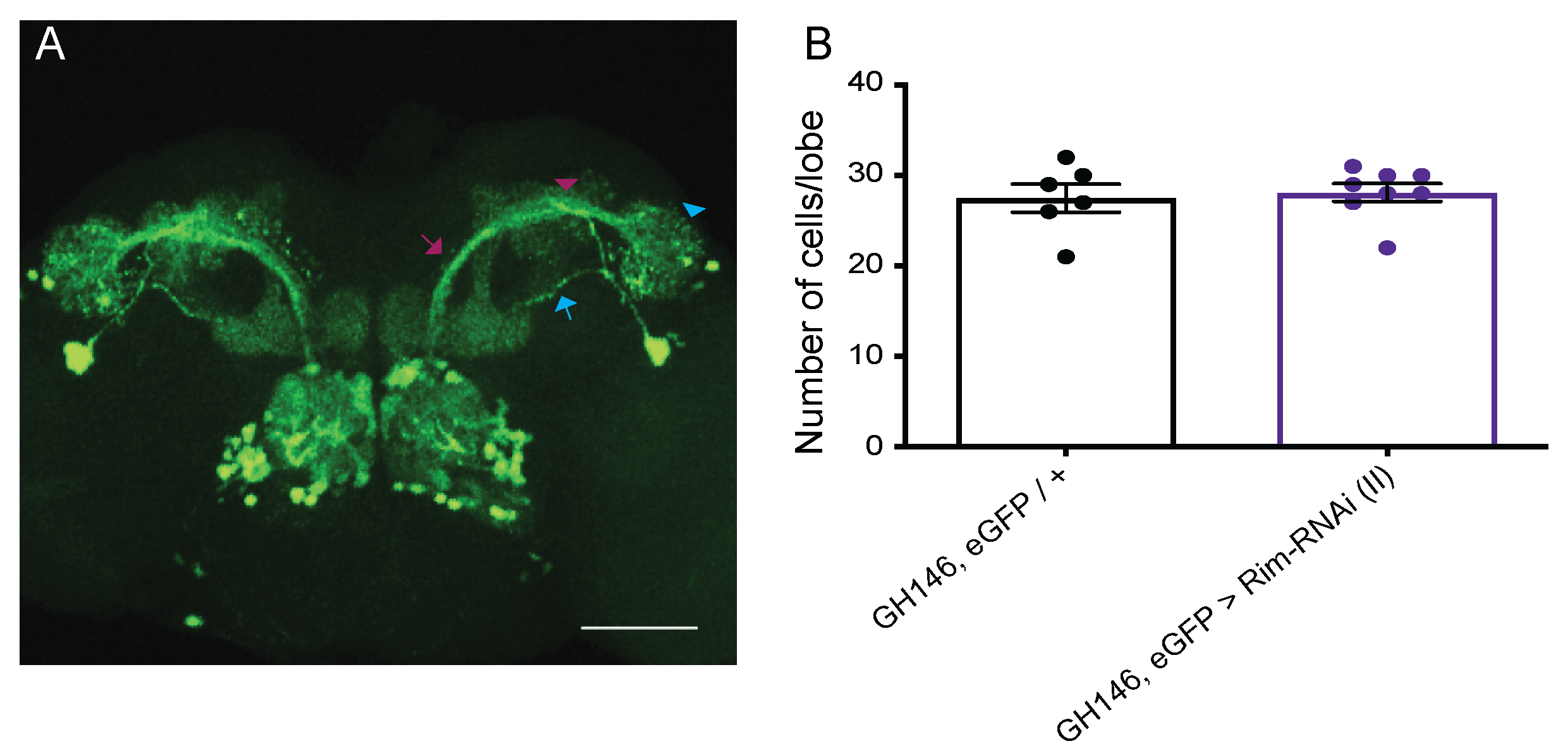
**

**Fig. S3.** *Rim* knock-down did not affect the number of antennal lobe projection neurons. A, Enhanced green fluorescent protein (eGFP) was expressed in the antennal lobe projection neurons (ALPNs) using the *GH146-GAL4* driver. ALPNs send projections to the mushroom bodies (dark pink arrowhead) and lateral horn (light blue arrowhead), through the inner antenno-cerebral tract (iACT, dark pink arrow) as well as directly to the lateral horn through the median antenno-cerebral tract (mACT, light blue arrowhead). Scale bar is 50 μm. B, The total number of eGFP positive neurons labelled with the GH146 driver was quantified per lobe. No differences were found when comparing *GH146, eGFP / +* control flies (black open bar) compared to *GH146, eGFP > Rim-RNAi* (II) (purple open bar) (Mann-Whitney test).

**
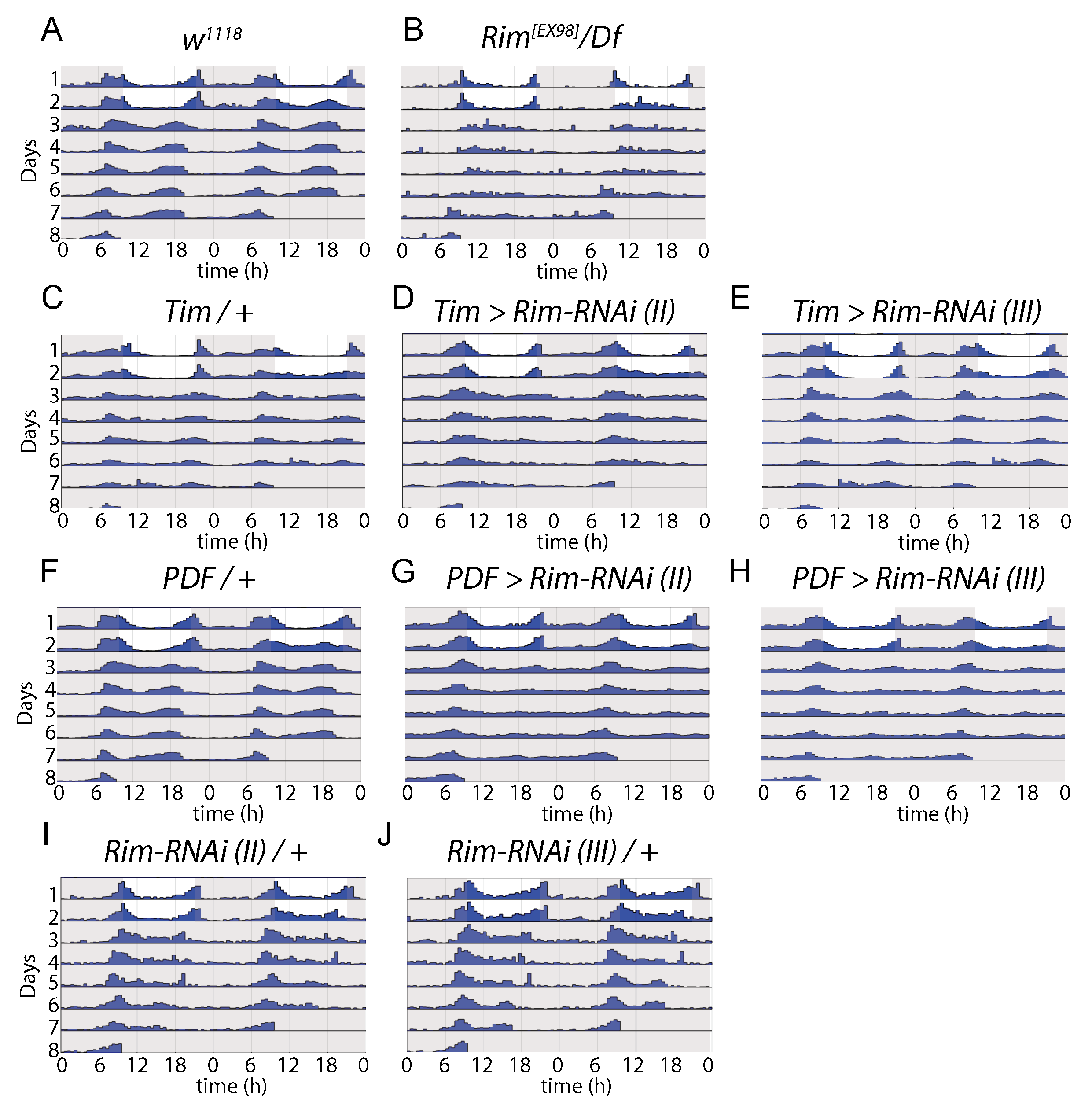
**

**Fig. S4.** *Rim* knockdown in the clock circuit affected rhythmic behaviour under constant darkness. Averaged double-plotted actograms of single flies of the following genotypes: A, *w^1118^*; B, *Rim^Ex98^/Df*; C, *Tim* / +; D, *Tim > Rim-RNAi (II)*; E, *Tim > Rim-RNAi (III)*; F, *PDF* / +; G, *PDF > Rim-RNAi (II)*; H, *PDF > Rim-RNAi (III);* I, *Rim-RNAi (II) / +* and J, *Rim-RNAi (III) / +*, for 2 days in LD (alternated white and grey in actograms) followed by 5 days in DD (grey). The activity patterns of *Rim^Ex98^/Df, Tim > Rim-RNAi* and *PDF > Rim-RNAi* flies appeared less rhythmic under DD than controls.

Table S2. The effect of clock neuron *Rim* knock-down on circadian behavioural parameters under constant darkness (DD).

| genotype | R.S. | % rhythmic | period (h) | n |
| --- | --- | --- | --- | --- |
| *w^1118^* | 3.68 ± 0.09 | 100 | 23.53 ± 0.03 | 29 |
| *Rim^Ex98^ / Df* | 1.29 ± 0.12 | 42 | 23.25 ± 0.14 | 12 |
| *Tim / +* | 2.45 ± 0.09 | 85 | 24.04 ± 0.05 | 82 |
| *Tim > Rim-RNAi (II)* | 1.93 ± 0.10 | 54 | 23.82 ± 0.07 | 22 |
| *Tim > Rim-RNAi (III)* | 2.07 ± 0.10 | 57 | 23.69 ± 0.04 | 32 |
| *PDF / +* | 3.14 ± 0.11 | 100 | 23.71 ± 0.03 | 61 |
| *PDF > Rim-RNAi (II)* | 1.99 ± 0.11 | 66 | 23.62 ± 0.06 | 94 |
| *PDF > Rim-RNAi (III)* | 1.71 ± 0.13 | 51 | 23.29 ± 0.05 | 63 |
| *Rim-RNAi (II) / +* | 2.76 ± 0.14 | 90 | 23.62 ± 0.06 | 32 |
| *Rim-RNAi (III) / +* | 2.78 ± 0.11 | 100 | 23.52 ± 0.08 | 29 |

This table is related to Fig. 4 and 5. Data are mean ± SEM.

**
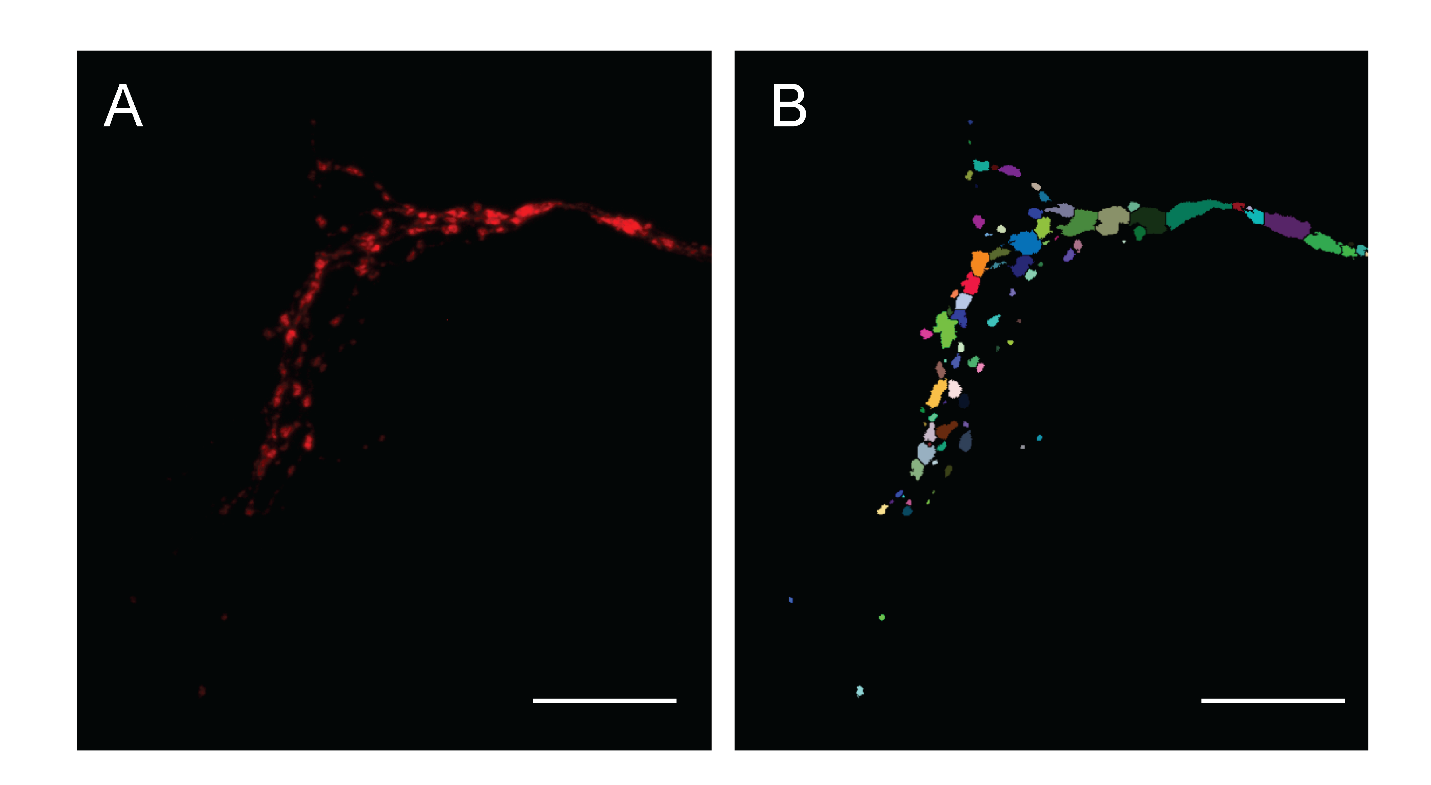
**

**Fig. S5.** Quantification of PDF intensity. Representative confocal images showing A, PDF immunohistochemical signal in the dorsal terminals of the s-LNvs and B, aggregates of PDF that were highlighted in different colours by an automatized image processing tool which obtained separation of each from its neighbours. Scale bars are 20 μm.
